# Altered somatosensory profile according to quantitative sensory testing in patients with degenerative lumbar spine disorders scheduled for surgery

**DOI:** 10.1186/s12891-017-1581-6

**Published:** 2017-06-17

**Authors:** Yvonne Lindbäck, Hans Tropp, Paul Enthoven, Björn Gerdle, Allan Abbott, Birgitta Öberg

**Affiliations:** 1 Department of Medical and Health Sciences, Division of Physiotherapy, Faculty of Medicine and Health Sciences, Linköping University, SE-581 83 Linköping, Sweden; 2 Department of Spinal Surgery and Department of Clinical and Experimental Medicine, Linköping University, Linköping, Sweden; 3 Center for Medical Image Science and Visualization (CMIV), Linköping University, Linköping, Sweden; 4 Pain and Rehabilitation Center, and Department of Medical and Health Sciences, Linköping University, Linköping, Sweden; 5 Faculty of Health Science and Medicine, Bond University, Gold Coast, QLD 4229 Australia; 6 http://www.imh.liu.se/fysioterapi

**Keywords:** Disc herniation, Spinal stenosis, Spondylolisthesis, Degenerative disc disease, Spine surgery, Quantitative sensory testing, Outcome

## Abstract

**Background:**

Somatosensory profiling in affected and non-affected body regions can strengthen our insight regarding the underlying pain mechanisms, which can be valuable in treatment decision making and to improve outcomes, in patients with degenerative lumbar spine disorders pre-surgery. The aim was to describe somatosensory profiles in patients with degenerative lumbar spine disorders, to identify the proportion with altered somatosensory profile, and to analyze demographic characteristics, self-reported function, pain, and health pre- and 3 months post-surgery.

**Methods:**

In this prospective cohort study in a Spine Clinic, 105 patients scheduled for surgery for spinal stenosis, disc herniation, degenerative disc disease, or spondylolisthesis were consecutively recruited. Exclusion criteria were; indication for acute surgery or previous surgery at the same spinal level or severe grade of pathology. Quantitative sensory testing (QST) and self-reported function, pain, and health was measured pre- and 3 months post-surgery. The somatosensory profile included cold detection threshold, warmth detection threshold, cold pain threshold, heat pain threshold and pressure pain threshold in affected and non-affected body regions.

**Results:**

On a group level, the patients’ somatosensory profiles were within the 95% confidence interval (CI) from normative reference data means. On an individual level, an altered somatosensory profile was defined as having two or more body regions (including a non-affected region) with QST values outside of normal ranges for reference data. The 23 patients (22%) with altered somatosensory profiles, with mostly loss of function, were older (*P* = 0.031), more often female (*P* = 0.005), had higher back and leg pain (*P* = 0.016, 0.020), lower mental health component summary score (SF-36 MCS) (*P* = 0.004) and larger pain distribution (*P* = 0.047), compared to others in the cohort. Post-surgery there was a tendency to worse pain, function and health in the group with altered somatosensory profile pre-surgery.

**Conclusions:**

On a group level, patients with degenerative lumbar spine disorders scheduled for surgery were within normal range for the QST measurements compared to reference values. On an individual level, an altered somatosensory profile outside of normal range in both affected and non-affected body regions occurred in 22% of patients, which may indicate disturbed somatosensory function. Those patients had mostly loss of sensory function and had worse self-reported outcome pre-surgery, compared to the rest of the cohort. Future prospective studies are needed to further examine whether these dimensions can be useful in predicting post-surgery outcome and guide need of additional treatments.

## Background

Among patients scheduled for spinal surgery due to degenerative lumbar spine disorders (e.g., disc herniation, spinal stenosis, spondylolisthesis, or degenerative disc disease), a majority have experienced pain for over three months. Persistent pain results from a complex interplay of many factors. Maladaptive neuroplastic changes in the central nervous system can be influenced by orthopedic structural pathology, as well as biochemical and psychosocial factors [1]. Such changes can result in increased pain sensitivity, and alterations of somatosensory or sensory function leading to amplified responses to nociception from localized spinal pathology, reduced pain thresholds, or widening distribution of pain [1].

In patients with low back pain (LBP), bedside neurological testing is recommended [2]. However, quantitative sensory testing (QST) can provide more specific and quantifiable somatosensory or sensory profiling [3]. QST assesses the function of; small myelinated A-delta fibers that conduct cold sensations and deep pain sensitivity, small unmyelinated C-fibers that conduct warmth, heat-pain sensations and deep pain sensitivity [4], and large A-beta fibers for light touch [3]. Perception in response to mechanical, thermal, or electrical stimulation at a controlled intensity [4, 5] can therefore be used to test sensory detection, pain thresholds, and pain summation [3].

QST can detect gain of sensory function in the form of lowered sensory thresholds (e.g., hyperesthesia and hyperalgesia) and loss of sensory function (e.g., hypoesthesia and hypoalgesia) [3, 4]. Through QST profiling in both affected and non-affected body regions, LBP symptoms can be more thoroughly assessed to determine the extent of localized hypoesthesia and hyperalgesia, due to increased nociceptive input in affected body region [6] or generalized hyperalgesia when there is alteration even in other body regions (arm and leg) not affected by LBP [7]. In patients with disc herniation, generalized deep tissue hyperalgesia has been reported using QST on the infraspinatus and anterior tibialis muscles [8]. Hypoesthesia to thermal detection in affected dermatome was reported among patients with sciatica [9, 10] and in patients with disc herniation scheduled for surgery, one study with 21 participants has reported both hypoesthesia and hypoalgesia in affected dermatome [11]. There is a lack of data about somatosensory function in patients with degenerative lumbar spine disorders, as for instance spinal stenosis or disc herniation, which represent the two largest groups that undergo spinal surgery [12]. Somatosensory profiling in both affected and non-affected body regions together with analysis of other biopsychosocial factors can strengthen our insight regarding the underlying pain mechanisms, which may help to guide the pre-surgical clinical decision-making process and need of additional management that may improve surgical outcomes [5]. The aim was to describe somatosensory profiles in patients with degenerative lumbar spine disorders, to identify the proportion with altered somatosensory profile, and to analyze demographic characteristics, self-reported function, pain, and health pre- and 3 months post-surgery.

## Methods

This prospective cohort study with cross sectional and prospective analysis investigated pre-surgery sensory profiles and biopsychosocial factors. The study conforms to the STROBE statement checklist for cohort studies. The patients received oral and written information about the study 1week before the measurements. All participants provided signed consent at the time of QST measurements. The study was approved by the Regional Ethics Committee (Dnr 2013/410-31).

### Participants and settings

A total of 105 patients were consecutively recruited at a Spine Clinic at a University Hospital, in Sweden, between September 2013 and December 2014. Inclusion criteria were; patients scheduled for surgery due to: disc herniation, spinal stenosis, spondylolisthesis (Grade 4) or degenerative disc disease, age between 25 and 80 years and fluent in Swedish. Exclusion criteria were; indication for acute surgery, previous surgery at the same spinal level or severe grade of pathology. Seventeen eligible patients chose not to participate due to the requirement of additional appointments at the hospital for QST.

### Procedure for QST measurements

Sensory profile investigation included the following QST measurements: cold detection threshold (CDT), warmth detection threshold (WDT), cold pain threshold (CPT), heat pain threshold (HPT), and pressure pain threshold (PPT). A standardized QST protocol [13, 14] was applied for all patients. CDT and WDT, and subsequently CPT and HPT, were measured using a thermic stimulator (Somedic, Hörby, Sweden). A thermode containing a Peltier element with a stimulating area of 25 × 50 mm was used. CDT, WDT, CPT, and HPT reportedly show a high degree of repeatability in healthy subjects [15], and acceptable repeatability in patients with sciatica [10]. For thermal tests, the baseline temperature was 32 °C, and the temperature was decreased or increased at a rate of 1 °C/s within a range of 10–50 °C. During the thermal measurements, the thermode was held on the test site. When measuring CDT or WDT, the patients were instructed to push a stop button when they first perceived a decreasing temperature or an increasing temperature, respectively. For CPT or HPT measurements, respectively, patients were instructed to push the stop button when the cold or heat sensation was first perceived as painful [13, 14]. Thermal measurements were performed on the following seven body regions: lower back (2 cm lateral of the spinal column on the most painful side, i.e., the symptomatic side), thighs (lower part of quadriceps muscle, 7–10 cm above the patella upper border, bilaterally), and lower legs (upper part of tibialis anterior muscle, 7–10 cm below the patella lower border, bilaterally) and two non-affected body-regions according to the degenerative lumbar spine disorders; hand (thenar eminence muscle on the dominant hand) and upper back (on the lower thoracic spine, contralateral to the lower back region). The two non-affected body regions were added to detect if there were patients with generalized alteration in sensory profile. Each thermal measurement was repeated five times, and the mean value was calculated for each patient.

PPT was measured using a handheld electrical pressure algometer (Somedic, Hörby, Sweden) with a 1-cm diameter probe. The patient was instructed to state when the pressure started to become painful, at which point the applied pressure was released [13, 14]. Pressure was applied at a rate of 30 kPa/s up to a maximal pressure of 700 kPa. PPT was measured once at each of five body regions, which included the same body regions used for thermal measurements, excluding the two spinal regions.

QST measurements were performed 1 to 2 weeks prior to surgery by a single investigator, a physiotherapist working at the Spine clinic. During QST measurements, the patients were comfortably seated or lying down in a quiet room with an air temperature of 22 °C. At the start of testing, patients were asked to use a visual analogue scale (VAS) to rate the average pain intensity in their back and legs during the last 2 weeks, as well as their current pain intensity at rest [16]. The patient’s most symptomatic side in the back or leg was also registered. The patients reported whether they had been able to refrain from using any stronger analgesics during the 24 h prior to QST, as had been recommended. In cases of analgesic use, the dosage was documented. Each test was initially performed on the non-dominant hand, with the purpose of familiarizing the patient with the QST protocol. These tests were not included in the analysis. Subsequently, the thermal sensory measurements were performed and finally the PPT measurements.

### Evaluation of function, pain, and health

To collect demographics and data regarding function, pain, and health pre-surgery the patients completed the questionnaire from the Swedish National Spine Register for spinal surgery patients (SweSpine) [12] and complementary questionnaires, including pain drawing [17], the Hospital Anxiety and Depression Scale (HADS) [18], the Self-Efficacy Scale (SES) [19], the fear avoidance beliefs questionnaire (FABQ) [20], and questions about lifestyle habits and expectations. Data regarding function, pain and health (EQ-5D, HADS and SES) was also collected 3 months post-surgery.

Function was measured using Oswestry Disability Index (ODI) [21], which includes ten items related to different functions and back pain, with six answer options (0–5) for each item, generating a sum score of between 0–100% disability [21]. ODI is the most commonly used instrument for this purpose [22, 23]. The patients rated their pain intensity the last week in the back and legs using a VAS with a horizontal line of 0–100 mm containing endpoints named “no pain” and “worse imaginable pain” [16]. Patients also reported their pain duration in the back and legs, with responses including “I don’t have pain”, “less than 3 months”, “3 to 12 months,” “1 to 2 years,” and “more than 2 years”. A pain drawing was used to identify whether patients had unilateral, bilateral, or no leg pain, as well as pain distribution in other body regions [17]. ODI and VAS pain are recommended instruments for measuring function and pain, respectively, in chronic LBP [22] and after spine surgery [24], with respect to validity and responsiveness [22, 24].

Health-related quality of life was measured using the European Quality of Life instrument (EQ-5D) [25, 26], which includes two scales: EQ-index and EQ-VAS. EQ-index includes five dimensions; mobility, self-care, usual activities, pain/discomfort, and anxiety/depression. Each dimension receives a score of 1–3, based on three possible answer options: “no problems”, “some problems”, “extreme problems”. The final EQ-index ranges from −0.594 to 1, with a higher score indicating better health status. The EQ-VAS is a 20 cm vertical scale ranging from a score of 0 indicating the “worst imaginable health state” (score 0) to a score of 100 indicating the “best imaginable health state”. EQ-5D is one of the five most commonly used questionnaires to measure health-related quality of life [22]. Mean EQ-5D index has been reported as 0.86 in a population in the UK, 0.84 in a Swedish population, and 0.66 among responders in the Swedish population with LBP [27].

Health was also measured with the Short Form Health Survey (SF-36) [28], which includes eight multi-items scales: bodily pain; vitality; general mental health; general health perceptions; limitations in physical functioning; limitations in usual role activities due to physical health; limitations in usual role activities due to personal and emotional problems; and limitations in social functioning due to physical or mental health problems. The subscales are summarized as physical and mental health component summary scores (PCS and MCS, respectively), each ranging from 0–100 with a higher score indicating better health [28]. Studies in a general population in Sweden show that the SF-36 has satisfactory reliability, construct-based validity, [29, 30] and criterion-based validity [31]. Another study reported that the EQ-5D, SF-36 PCS, and SF-36 MCS show a medium responsiveness after lumbar surgery, while the SF-36 total score shows low responsiveness [24].

Symptoms of depression and anxiety was measured using the Hospital Anxiety and Depression Scale (HADS) [18], which includes seven items for anxiety and seven items for depression. The HADS total score ranges from 0–21, with a higher score indicating more signs of anxiety or depression. The Swedish version of HADS is reportedly a robust instrument with regard to reliability, discriminant validity, concurrent validity, and ability to be a case finder for anxiety and depression [32].

The Self-Efficacy Scale (SES) is a 20-item scale for assessing a patient’s confidence regarding activities of daily living [19]. The scores range from 0 indicating “not at all confident” to 10 indicating “very confident”, with a higher score indicating better self-efficacy. The English version was developed for use in patients with LBP, and shows good internal consistency [19]. The Swedish version has been modified to be suitable for use in patients with all kinds of pain [33], shown good internal consistency [34] and test-retest reliability among patients with whiplash-associated disorder (WAD) [35].

Fear avoidance was measured using FABQ [20], which is a 16-item questionnaire focused on a patient’s beliefs. FABQ questions comprise a 4-item subscale describing how physical activity affects the patient’s pain (FABQ-PA). Each item is answered on a 7-grade scale, where a higher number indicates a higher level of fear avoidance beliefs. The English version of FABQ shows good test-retest reliability and internal consistency in patients with chronic LBP [20], and the Swedish version shows good internal consistency among patients with whiplash-associated disorder [34].

### Statistical methods

Statistical analysis was performed using IBM SPSS statistics version 23. The level of significance was set to *P* = 0.05.

Sample size calculation: Using CPT, HPT and PPT as outcomes at least 23 patients in each group were required based on calculations from a previous study that compared healthy subjects and subjects with chronic WAD [14]. For comparison between two groups of patients at least 17 patients in each group was required, based on sample size calculation in two subgroups of patients with WAD [13]. Sample size calculations were done using the computer program Power and Sample Size Calculations, version. 3.0.43 Vanderbilt University, US [36].

Patient demographics were analyzed using descriptive statistics, and are presented as mean and SD for continuous variables, and as frequencies and percentages for categorical variables. For between-group comparisons of demographic data, function, pain, and health, the unpaired Student’s *t*-test or Mann–Whitney *U* test for continuous variables, and the Chi-Square test or Fisher Exact probability test for categorical variables were used. In the *t*-test, if Levine’s test was significant, the *P* value for “equal variance not assumed” was reported.

The data in the sensory profiles data were compared with reference data, obtained of healthy subjects from studies of the German Research Network on Neuropathic Pain (DFNS) [37, 38]. QST data for the hand, thigh, and lower leg were compared with reference data for hand and feet from Magerl et al. [37]. QST data regarding the upper and lower back were compared with reference data reported by Pfau et al. [38]. CDT, WDT, and PPT were log-transformed. To compare the patient’s sensory profile with reference data, all QST data were standardized with *Z-*transformation, meaning that each QST value was matched for age and sex in the reference values [3]. The following expression was used for *Z*-transformation:

$$ Z\hbox{--} score = \left({X}_{single\  patient}\hbox{--}\ Mea{n}_{references}\right)/ S{D}_{references}. $$

A *Z*-score of < −2 or >2 is outside of the 95% confidence interval (CI) of a normal standard distribution for healthy subjects [39]. Moreover, *Z*-score values of <0 indicate a loss of sensory function, while *Z*-score values of >0 indicate r a gain of sensory function [39]. For the comparison between symptomatic and non-symptomatic body regions in the legs, as identified by the pain drawing, only the patients with unilateral leg pain were included.

An altered somatosensory profile was defined as having two or more body regions including a normally non-affected body region, i.e., the hand and/or upper back—with a *Z*-score of < −2 or >2 compared to reference data for CDT, WDT, CPT, HPT, or PPT.

## Results

### Demographics

Table 1 presents demographic data and self-reported function, pain, and health for the 105 patients. The mean age was 59.8 ± 12.9 years, and the cohort included 55 women (52%). The represented degenerative lumbar spine disorders included spinal stenosis (*n* = 61; 58.1%), disc herniation (*n* = 30; 28.6%), degenerative disc disease (DDD; *n* = 8; 7.6%), and spondylolysis/spondylolisthesis (*n* = 6; 5.7%). Patients with spinal stenosis were significantly older (67.6 ± 7.6 years) than patients with disc herniation (47.7 ± 11.4 years; *P* < 0.001). A pain duration of > 2 years was reported by 55 patients (52.5%), and was significantly more common among patients with spinal stenosis compared to patients with disc herniation (*P* = 0.003).

**Table 1:** Pre-surgery measures for all patients, comparison between those with spinal stenosis and disc herniation

	All patients *n* = 105^a^		Spinal stenosis *n* = 61^b^		Disc herniation *n* = 30^c^		Spinal stenosis/Disc herniation *P*
Age, mean (SD)	59.8	(12.9)	67.6	(7.6)	47.7	(11.4)	<0.001
Women, *n* (%)	55	(52.4)	32	(52.5)	16	(53.3)	0.937
Pain duration back/leg > 2 years, *n* (%)	55	(52.5)	38	(63.3)	8	(30.7)	0.003
ODI, mean (SD)	38.4	(15.6)	37.9	(15.3)	40.2	(16.0)	0.539
VAS back pain, mean (SD)	53.8	(26.4)	54.1	(25.0)	49.9	(28.8)	0.493
VAS leg pain, mean (SD)	61.2	(25.2)	61.9	(22.0)	67.4	(24.3)	0.298
EQ-5D index, mean (SD)	0.42	(0.31)	0.41	(0.32)	0.41	(0.29)	0.924
EQ-VAS, mean (SD)	50.5	(21.8)	51.4	(21.6)	50.7	(22.0)	0.896
HADS anxiety, mean (SD)	6.0	(3.8)	5.6	(3.5)	5.9	(4.1)	0.744
HADS depression, mean (SD)	4.7	(3.3)	4.3	(3.1)	4.5	(3.5)	0.773
SES, mean (SD)	129.8	(40.8)	132.5	(40.3)	125.1	(44.0)	0.451
SF-36 PCS, mean (SD)	29.4	(9.1)	28.5	(9.4)	30.3	(8.9)	0.417
SF-36 MCS, mean (SD)	46.4	(12.5)	47.9	(11.4)	45.5	(15.0)	0.419
FABQ-PA, mean (SD)	14.8	(6.0)	14.0	(6.3)	15.5	(5.6)	0.321
Pain drawing *n* (%):							
Back and/or unilateral leg pain	41	(39.8)	14	(23.35)	19	(65.5)	<0.001
Bilateral leg pain	43	(41.7)	34	(56.7)	6	(20.7)	
Back- leg pain and other pain locations	19	(18.4)	12	(20.0)	4	(13.8)	

*SD* standard deviation, *ODI* Oswestry Disability Index (0–100) (higher score indicate higher disability), *VAS* visual analogue scale (0–100) (higher score indicate higher pain intensity); *EQ-5D (− 0.594 - 1) and EQ-VAS* EuroQol (higher score indicate better health), *HADS* Hospital anxiety and depression scale (0–21) (higher score indicate more signs of symptoms), *SES* Self-Efficacy Scale (0–200) (higher score indicate better self-efficacy), *SF-36 PCS* physical component summery and *MCS* mental component summery (0–100) (higher score indicate better health), *FABQ-PA* fear avoidance beliefs questionnaire – physical activity (0–24) (higher score indicates higher level of fear avoidance beliefs)

^a^Maximum missing data in each column: *n* = 11, ^b^ *n* =9, ^c^ *n* = 4

### Somatosensory profiles of patients on a group level compared to reference data

**Fig. 1:**
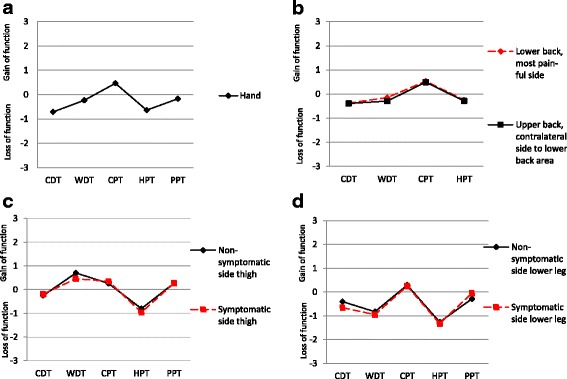
Somatosensory profile of patients compared to reference data. Somatosensory profiles from the hand (**a**) (*n* = 105), upper and lower back (**b**) (*n* = 105), thigh (**c**) (*n* = 48), and lower leg (**d**) (*n* = 48). *Z*-scores were calculated to standardize the study population values according to the mean and SD from the reference data [37, 38]. A *Z*-score of >0 indicates a gain of function where the patient is more sensitive, while a Z-score of <0 indicates a loss of function where the patient is less sensitive to test stimuli compared to controls. A *Z*-score of between −2 and 2 indicates that data is within the 95% confidence interval of the normal standard distribution [39]. CDT, cold detection threshold; WDT, warm detection threshold; CPT, cold pain threshold; HPT, heat pain threshold, PPT, pressure pain threshold

On a group level the somatosensory profiles of the patients showed that all QST measurements had mean *Z*-score values between −2 and 2, meaning that the group values were within the 95% CI of the reference data (Fig. 1). The Z-scores for CDT and HPT were below “0” in all body regions tested. The Z-scores for WDT were below “0” in all body regions except for symptomatic and non-symptomatic thigh. Furthermore, the Z-scores for CPT were above “0” in all body regions tested. Forty-eight patients had unilateral pain with no significant differences between symptomatic and non-symptomatic side in the thigh or lower leg (Fig. 1).

### Proportion of patients on an individual level with altered somatosensory profile

A total of 23 patients had altered somatosensory profiles for at least one QST measurement in the hand or upper back and at least one additional body region (Table 2). Compared to the other 82 patients, the patients with altered somatosensory profiles had a significantly higher mean age. Additionally, more women than men had an altered somatosensory profile. The two groups showed no significant differences in the proportions of patients with spinal stenosis and disc herniation, or the proportions of patients with pain duration of greater than 2 years.

**Table 2:** Pre- and post-surgery measurements, comparison between those with and without altered sensory profile in QST

	Pre-surgery measurements					Post-surgery measurements				
	Without altered sensory profile *n* = 82^a^		Altered sensory profile * n* = 23^b^		*P*	Without altered sensory profile *n* = 79^c^		Altered sensory profile *n* = 21^d^		*P*
Age, mean (SD)	58.6	(12.5)	64.0	(13.8)	0.031					
Women, *n* (%)	37	(45)	18	(78)	0.005					
Spinal stenosis, *n* (%)	45	(55)	16	(70)	0.207					
Disc herniation, *n* (%)	24	(29)	6	(26)	0.765					
Pain duration >2 years, n (%)	44	(56)	11	(50)	0.859					
ODI, mean (SD)	37.1	(15.9)	42.9	(15.7)	0.112	28.4	(17.2)	30.8	(18.0)	0.566
VAS back pain, mean (SD)	50.9	(27.5)	63.6	(19.3)	0.016	28.8	(26.2)	38.3	(26.6)	0.147
ODI > 40% *n* (%)	37	(48.1)	15	65.2)	0.148	14	(17.9)	6	(28.6)	0.641
VAS leg pain, mean (SD)	58.8	(27.0)	69.3	(15.1)	0.020	23.7	(28.5)	34.0	(27.5)	0.146
EQ-5D index, mean (SD)	0.43	(0.30)	0.37	(0.32)	0.430	0.62	(0.28)	0.68	(0.16)	0.356
EQ-VAS, mean (SD)	51.9	(22.2)	45.9	(21.1)	0.250	66.3	(21.3)	73.4	(19.7)	0.173
HADS anxiety, mean (SD)	5.8	(3.6)	6.6	(4.49)	0.345	4.05	(3.1)	5.4	(4.0)	0.110
HADS depression, mean (SD)	4.6	(3.2)	4.8	(3.9)	0.832	3.27	(2.93)	3.84	(3.30)	0.455
SES, mean (SD)	132.9	(41.5)	116.3	(35.4)	0.120	155.0	(32.7)	146.2	(41.9)	0.348
SF-36 PCS, mean (SD)	30.1	(9.4)	26.9	(7.3)	0.117					
SF-36 MCS, mean (SD)	48.3	(11.69)	39.8	(13.06)	0.004					
FABQ-PA, mean (SD)	14.8	(6.1)	15.0	(6.0)	0.870					
Pain drawing *n* (%):										
Back or unilateral leg pain	37	(46.3)	4	(17.4)	0.029					
Bilateral leg pain	31	(38.8)	12	(52.1)						
Back- leg pain and other pain locations	12	(15)	7	(30.4)						

*QST* Quantitative sensory testing, *SD* standard deviation, *ODI* Oswestry Disability Index (0–100) (higher score indicate higher disability), *VAS* visual analogue scale (0–100) (higher score indicate higher pain intensity); EQ-5D (− 0.594 - 1) and *EQ-VAS* EuroQol (higher score indicate better health), *HADS* Hospital anxiety and depression scale (0–21) (higher score indicate more signs of symptoms), *SES* Self-Efficacy Scale (0–200) (higher score indicate better self-efficacy), *SF-36 PCS*, physical component summery and MCS, mental component summery (0 – 100) (higher score indicate better health), *FABQ-PA* fear avoidance beliefs questionnaire – physical activity (0–24) (higher score indicates higher level of fear avoidance beliefs)

^a^Maximum missing data in each column: *n* = 7, ^b^ *n* =4, ^c^ *n* = 6, ^d^ *n* = 4

Of the 23 patients with altered somatosensory profiles, 19 (83%) had *Z*-scores of < −2 in one of the QST measurements in at least two body regions compared to the reference data, indicating that they had loss of sensory function. The remaining 4 patients with altered somatosensory profiles showed *Z*-scores of >2 compared to references data, indicating that they had a gain of sensory function and were more sensitive for one test in at least two body regions. *Z-*scores of < −2 or >2 were observed in all QST measurements (CDT, WDT, CPT, HPT and PPT), except for Z-scores of < −2 in CPT.

#### Function, pain, and health in patients with or without altered somatosensory profile before and after surgery

Compared to patients without an altered somatosensory profile, those with an altered somatosensory profile reported significantly higher pre-surgery back and leg pain VAS and lower score on SF-36 MCS (Table 2). The post-surgery results showed no significant differences between the groups but there was a tendency towards worse function, pain and health in the group with altered somatosensory profile pre-surgery. Pain drawings pre-surgery showed that patients with altered somatosensory profile had larger distribution of pain than the rest of the cohort (*P* = 0.047).

## Discussion

In line with the study’s aim to investigate the proportion of individuals showing an altered somatosensory profile, 22% of patients were detected. These patients had alterations outside of normal range in two or more body regions, including a non-affected region. This can be considered a sign of generalized alteration of somatosensory function. Most of these patients had loss of sensory function. The group with altered somatosensory profile showed worse back and leg pain VAS and SF-36 MCS before surgery, and also had larger distribution of pain as reported in pain drawings pre-surgery. The 3 months post-surgery results with patient reported outcome measures could not confirm the pre-surgery differences between the group with- respectively without altered somatosensory function, but it showed a tendency with worse function, pain and health in the group with altered somatosensory profile before surgery. The study does not have the power to detect small differences since the group with altered somatosensory profile turned out to be small. Considering that 20–35% of the patients with spinal stenosis and disc herniation are doubtful or dissatisfied with the results at 1-year follow up post-surgery [12], it still needs to be proven if the use of a somatosensory profile could be helpful to understand effects of treatment in larger studies.

When considering the other study aim, to describe somatosensory profiles of patients on a group level, Z-scores for QST data compared to reference data showed that our cohort was within the 95% CI of the reference values [37, 38]. In many of the QST measurements, the patient group had a tendency to loss of sensory function (Z-score below “0”), while CPT showed a tendency to gain of sensory function (Z-score above “0”) in all body regions tested, which means that the patient group had a tendency to be more sensitive to cold pain than the reference data.

The choice of QST measurements is of importance and in subjects with persistent pain, thermal pain thresholds are of greater importance than detection thresholds [40]. People are more sensitive to cold than warmth, partly because the receptors for cold are more superficially located and are present in larger amounts compared to receptors for warmth [41]. With regard to CPT, this patient cohort showed a tendency to gain of function (greater sensitivity) in all body regions compared to reference data. Greater sensitivity to cold could indicate disturbed sensory function [3]. Roussel et al. [42] reported conflicting results concerning responsiveness to various stimuli including different aspects of sensory testing in patients with chronic LBP. QST measurement in chronic nonspecific LBP divided into mechanical pain and non-mechanical pain showed higher odds for cold hyperalgesia in the non-mechanical than in the mechanical LBP group [43]. Also patients with acute non-specific LBP have shown increased sensitivity to cold pain as well as to mechanical stimuli in comparison to pain-free controls [44]. In patients with fibromyalgia higher sensitivity in CPT was associated with higher pain intensity, more tender points, and poorer sleep [40]. Beside CPT, PPT has been suggested as the most promising QST measurement to discriminating pain in osteoarthritis [45] and PPT and electrical pain detection thresholds in chronic LBP [46]. In patients scheduled for hip- or knee replacement, higher sensitivity to PPT in a non-affected body region was associated with higher pain intensity [47]. In the current study on group level, Z-scores for PPT for all body regions were close to “0”, indicating that there were no differences in PPT compared to reference values. Studies point out that QST [48] or cold sensitivity [43] is one part of an examination, and should be seen together with other findings to further study the effects of different treatments.

Patients with altered somatosensory profile had mostly loss of sensory function in current study. Similar result with loss of sensory function pre-surgery has previous been presented in patients with disc herniation [11], that study also reported that complete recovery after surgery was associated with normalization of QST. Another study reported a trend towards higher loss of sensory function in QST in patients with higher degree of nerve root compression in a cohort of patients with MRI verified disc herniation [49]. The patients with altered somatosensory profile in the current study were older and included more women, compared to the rest of the cohort, even though each individual QST measurement was compared to age and gender adjusted reference data in the *Z*-score. Therefore, we suggest that the different profile in this particular group is related to other factors, which can be potential mediators of outcomes after treatment. Associations between sensory profiles and psychological variables have also been reported in studies of non-specific LBP [50], WAD [14], and fibromyalgia [40]. To our knowledge, our present study is the first investigation of patients with degenerative lumbar spine disorders prior to surgery to report differences in pain intensity, mental health components and pain distribution in pain drawings between patients with or without altered sensory profiles.

Generalizability is one important issue. The questionnaires used are internationally recommended self-reported questionnaires of function, pain, and health providing a thorough biopsychosocial description of the patient cohort. In the study, all patients are screened by an orthopedic surgeon and assessed as suitable candidates for surgery, in a shared decision with the patient. The population is representative of the population scheduled for surgery in Sweden when compared to the population included in the national registry SweSpine with regard to the proportions of different diagnoses, ages, genders, functions, and pain [12]. Additionally, in the present study, all tests were performed by one independent physiotherapist, potentially strengthening reliability of the testing procedure.

It has been reported that QST may have predictive value for identifying allodynia and hyperalgesia [48], but comparison with reference data is necessary for decision-making. In the current study, the best existing reference data was used, the DFNS reference data [37, 38]. However, this reference data has a broad range of variation in normal values — especially for CPT [37, 38], which may limit the possibility of identifying patients with altered sensory profiles.

One limitation in the study was, in the protocol used in this study tests for cold stopped at +10°, while DFNS used 0°, indicating a risk for underestimation rather than overestimation of the results. When CPT in reference data had the broad normal range 32–0 °C in most age groups [37, 38], that difference in degrees at end-points had minor influence on the result. Another limitation was that as there were no reference data available for thigh and lower leg, reference data for the foot were used instead [37]. Although this was not optimal, CPT and HPT measurements are generally quite uniform among different body regions [3]. Moreover, PPT measured on muscle tissue shows less variability between body regions compared to measurements on bone or nailbed tissue [51]. PPT measurements on thigh and lower leg were on muscle tissue, suggesting minimal influence of variability regarding the use of foot reference data.

## Conclusions

On a group level, patients with degenerative lumbar spine disorders scheduled for surgery were within the normal range for the QST measurements compared to age and gender adjusted reference values. This might be interpreted as a well-selected patient group for surgery according to QST. However, on an individual level, 22% of the patients showed altered somatosensory profiles, when defined as alterations in two or more body regions, including a non-affected region, which may indicate disturbed somatosensory function. These patients had mostly loss of sensory function and reported worse pain and mental health components as well as larger pain distribution pre-surgery compared to the rest of the cohort. Differences 3 months post-surgery could not be confirmed partly due to limited study power. It remains to be determined whether baseline QST measurements have predictive value, and whether change over time is important for the outcome following interventions.

## Abbreviations

QST: Quantitative sensory testing
LBP: Low back pain
CDT: Cold detection threshold
WDT: Warmth detection threshold
CPT: Cold pain threshold
HPT: Heat pain threshold
PPT: Pressure pain threshold

## References

[1] Woolf CJ (2011). Central sensitization: implications for the diagnosis and treatment of pain. Pain.

[2] Airaksinen O, Brox JI, Cedraschi C, Hildebrandt J, Klaber-Moffett J, Kovacs F, Mannion AF, Reis S, Staal JB, Ursin H (2006). Chapter 4. European guidelines for the management of chronic nonspecific low back pain. Eur Spine J.

[3] Rolke R, Magerl W, Campbell KA, Schalber C, Caspari S, Birklein F, Treede RD (2006). Quantitative sensory testing: a comprehensive protocol for clinical trials. Eur J Pain.

[4] Cruccu G, Anand P, Attal N, Garcia-Larrea L, Haanpaa M, Jorum E, Serra J, Jensen TS (2004). EFNS guidelines on neuropathic pain assessment. Eur J Neurol.

[5] Arendt-Nielsen L, Yarnitsky D (2009). Experimental and clinical applications of quantitative sensory testing applied to skin, muscles and viscera. J Pain.

[6] Blumenstiel K, Gerhardt A, Rolke R, Bieber C, Tesarz J, Friederich HC, Eich W, Treede RD (2011). Quantitative sensory testing profiles in chronic back pain are distinct from those in fibromyalgia. Clin J Pain.

[7] O’Neill S, Kjaer P, Graven-Nielsen T, Manniche C, Arendt-Nielsen L (2011). Low pressure pain thresholds are associated with, but does not predispose for, low back pain. Eur Spine J.

[8] O’Neill S, Manniche C, Graven-Nielsen T, Arendt-Nielsen L (2007). Generalized deep-tissue hyperalgesia in patients with chronic low-back pain. Eur J Pain.

[9] Freynhagen R, Rolke R, Baron R, Tolle TR, Rutjes AK, Schu S, Treede RD (2008). Pseudoradicular and radicular low-back pain--a disease continuum rather than different entities? Answers from quantitative sensory testing. Pain.

[10] Zwart JA, Sand T (2002). Repeatability of dermatomal warm and cold sensory thresholds in patients with sciatica. Eur Spine J.

[11] Zub LW, Szymczyk M, Pokryszko-Dragan A, Bilinska M (2013). Evaluation of pain in patients with lumbar disc surgery using VAS scale and quantitative sensory testing. Adv Clin Exp Med.

[12] Stromqvist B, Fritzell P, Hagg O, Jonsson B, Sanden B (2013). Swespine: the Swedish spine register: the 2012 report. Eur Spine J.

[13] Borsbo B, Liedberg GM, Wallin M, Gerdle B (2012). Subgroups based on thermal and pressure pain thresholds in women with chronic whiplash display differences in clinical presentation - an explorative study. J Pain Res.

[14] Wallin M, Liedberg G, Borsbo B, Gerdle B (2012). Thermal detection and pain thresholds but not pressure pain thresholds are correlated with psychological factors in women with chronic whiplash-associated pain. Clin J Pain.

[15] Heldestad V, Linder J, Sellersjo L, Nordh E (2010). Reproducibility and influence of test modality order on thermal perception and thermal pain thresholds in quantitative sensory testing. Clin Neurophysiol.

[16] Scott J, Huskisson EC (1976). Graphic representation of pain. Pain.

[17] Southerst D, Cote P, Stupar M, Stern P, Mior S (2013). The reliability of body pain diagrams in the quantitative measurement of pain distribution and location in patients with musculoskeletal pain: a systematic review. J Manip Physiol Ther.

[18] Zigmond AS, Snaith RP (1983). The hospital anxiety and depression scale. Acta Psychiatr Scand.

[19] Altmaier E (1993). Role of self-efficacy in rehabilitation outcome among chronic low back pain patients. J Couns Psychol.

[20] Waddell G, Newton M, Henderson I, Somerville D, Main CJ (1993). A Fear-Avoidance Beliefs Questionnaire (FABQ) and the role of fear-avoidance beliefs in chronic low back pain and disability. Pain.

[21] Fairbank JC, Couper J, Davies JB, O’Brien JP (1980). The Oswestry low back pain disability questionnaire. Physiotherapy.

[22] Chapman JR, Norvell DC, Hermsmeyer JT, Bransford RJ, DeVine J, McGirt MJ, Lee MJ (2011). Evaluating common outcomes for measuring treatment success for chronic low back pain. Spine.

[23] Ostelo RW, Deyo RA, Stratford P, Waddell G, Croft P, Von Korff M, Bouter LM, de Vet HC (2008). Interpreting change scores for pain and functional status in low back pain: towards international consensus regarding minimal important change. Spine.

[24] DeVine J, Norvell DC, Ecker E, Fourney DR, Vaccaro A, Wang J, Andersson G (2011). Evaluating the correlation and responsiveness of patient-reported pain with function and quality-of-life outcomes after spine surgery. Spine.

[25] EuroQolGroup (1990). EuroQol--a new facility for the measurement of health-related quality of life. Health Policy.

[26] Brooks R (1996). EuroQol: the current state of play. Health Policy.

[27] Burstrom K, Johannesson M, Diderichsen F (2001). Swedish population health-related quality of life results using the EQ-5D. Qual Life Res.

[28] Aaronson NK, Acquadro C, Alonso J, Apolone G, Bucquet D, Bullinger M, Bungay K, Fukuhara S, Gandek B, Keller S (1992). International Quality of Life Assessment (IQOLA) Project. Qual Life Res.

[29] Persson LO, Karlsson J, Bengtsson C, Steen B, Sullivan M (1998). The Swedish SF-36 Health Survey II. Evaluation of clinical validity: results from population studies of elderly and women in Gothenborg. J Clin Epidemiol.

[30] Sullivan M, Karlsson J, Ware JE (1995). The Swedish SF-36 Health Survey--I. Evaluation of data quality, scaling assumptions, reliability and construct validity across general populations in Sweden. Soc Sci Med.

[31] Sullivan M, Karlsson J (1998). The Swedish SF-36 Health Survey III. Evaluation of criterion-based validity: results from normative population. J Clin Epidemiol.

[32] Bjelland I, Dahl AA, Haug TT, Neckelmann D (2002). The validity of the hospital anxiety and depression scale. An updated literature review. J Psychosom Res.

[33] Denison E, Asenlof P, Lindberg P (2004). Self-efficacy, fear avoidance, and pain intensity as predictors of disability in subacute and chronic musculoskeletal pain patients in primary health care. Pain.

[34] Soderlund A, Olerud C, Lindberg P (2000). Acute whiplash-associated disorders (WAD): the effects of early mobilization and prognostic factors in long-term symptomatology. Clin Rehabil.

[35] Bunketorp L, Carlsson J, Kowalski J, Stener-Victorin E (2005). Evaluating the reliability of multi-item scales: a non-parametric approach to the ordered categorical structure of data collected with the Swedish version of the Tampa Scale for Kinesiophobia and the Self-Efficacy Scale. J Rehabil Med.

[36] Dupont WD, Plummer WD. Power and Sample Size Calculations. [http://biostat.mc.vanderbilt.edu/wiki/Main/PowerSampleSize]. Accessed 10 June 2017.

[37] Magerl W, Krumova EK, Baron R, Tolle T, Treede RD, Maier C (2010). Reference data for quantitative sensory testing (QST): refined stratification for age and a novel method for statistical comparison of group data. Pain.

[38] Pfau DB, Krumova EK, Treede RD, Baron R, Toelle T, Birklein F, Eich W, Geber C, Gerhardt A, Weiss T (2014). Quantitative sensory testing in the German Research Network on Neuropathic Pain (DFNS): reference data for the trunk and application in patients with chronic postherpetic neuralgia. Pain.

[39] Rolke R, Baron R, Maier C, Tolle TR, Treede RD, Beyer A, Binder A, Birbaumer N, Birklein F, Botefur IC (2006). Quantitative sensory testing: a comprehensive protocol for clinical trials. Eur J Pain.

[40] Hurtig IM, Raak RI, Kendall SA, Gerdle B, Wahren LK (2001). Quantitative sensory testing in fibromyalgia patients and in healthy subjects: identification of subgroups. Clin J Pain.

[41] Guergova S, Dufour A (2011). Thermal sensitivity in the elderly: a review. Ageing Res Rev.

[42] Roussel NA, Nijs J, Meeus M, Mylius V, Fayt C, Oostendorp R (2013). Central sensitization and altered central pain processing in chronic low back pain: fact or myth?. Clin J Pain.

[43] O’Sullivan P, Waller R, Wright A, Gardner J, Johnston R, Payne C, Shannon A, Ware B, Smith A (2014). Sensory characteristics of chronic non-specific low back pain: a subgroup investigation. Man Ther.

[44] Starkweather AR, Ramesh D, Lyon DE, Siangphoe U, Deng X, Sturgill J, Heineman A, Elswick RK, Dorsey SG, Greenspan J (2016). Acute Low back pain: differential somatosensory function and gene expression compared with healthy no-pain controls. Clin J Pain.

[45] Suokas AK, Walsh DA, McWilliams DF, Condon L, Moreton B, Wylde V, Arendt-Nielsen L, Zhang W (2012). Quantitative sensory testing in painful osteoarthritis: a systematic review and meta-analysis. Osteoarthr Cartil.

[46] Neziri AY, Curatolo M, Limacher A, Nuesch E, Radanov B, Andersen OK, Arendt-Nielsen L, Juni P (2012). Ranking of parameters of pain hypersensitivity according to their discriminative ability in chronic low back pain. Pain.

[47] Wylde V, Sayers A, Lenguerrand E, Gooberman-Hill R, Pyke M, Beswick AD, Dieppe P, Blom AW (2015). Preoperative widespread pain sensitization and chronic pain after hip and knee replacement: a cohort analysis. Pain.

[48] Backonja MM, Attal N, Baron R, Bouhassira D, Drangholt M, Dyck PJ, Edwards RR, Freeman R, Gracely R, Haanpaa MH (2013). Value of quantitative sensory testing in neurological and pain disorders: NeuPSIG consensus. Pain.

[49] Hegarty D, O’Conner OJ, Moore M, O’Regan KN, Shorten G, Maher M (2011). Association between preoperative magnetic resonance imaging, pain intensity and quantitative sensory testing in patients awaiting lumbar diskectomy. J Med Imaging Radiat Oncol.

[50] George SZ, Bialosky JE, Wittmer VT, Robinson ME (2007). Sex differences in pain drawing area for individuals with chronic musculoskeletal pain. J Orthop Sports Phys Ther.

[51] Rolke R, Andrews Campbell K, Magerl W, Treede RD (2005). Deep pain thresholds in the distal limbs of healthy human subjects. Eur J Pain.

